# A Two-Steps-Ahead Estimator for Bubble Entropy

**DOI:** 10.3390/e23060761

**Published:** 2021-06-16

**Authors:** George Manis, Matteo Bodini, Massimo W. Rivolta, Roberto Sassi

**Affiliations:** 1 Department of Computer Science and Engineering, University of Ioannina, 45500 Ioannina, Greece; 2 Dipartimento di Informatica, Università degli Studi di Milano, 20133 Milan, Italy; matteo.bodini@unimi.it (M.B.); massimo.rivolta@unimi.it (M.W.R.)

**Keywords:** entropy, bubble entropy, limited dependence on parameters

## Abstract

*Aims*: Bubble entropy (bEn) is an entropy metric with a limited dependence on parameters. bEn does not directly quantify the conditional entropy of the series, but it assesses the change in entropy of the ordering of portions of its samples of length *m*, when adding an extra element. The analytical formulation of bEn for autoregressive (AR) processes shows that, for this class of processes, the relation between the first autocorrelation coefficient and bEn changes for odd and even values of *m*. While this is not an issue, per se, it triggered ideas for further investigation. *Methods*: Using theoretical considerations on the expected values for AR processes, we examined a two-steps-ahead estimator of bEn, which considered the cost of ordering two additional samples. We first compared it with the original bEn estimator on a simulated series. Then, we tested it on real heart rate variability (HRV) data. *Results*: The experiments showed that both examined alternatives showed comparable discriminating power. However, for values of 10<m<20, where the statistical significance of the method was increased and improved as *m* increased, the two-steps-ahead estimator presented slightly higher statistical significance and more regular behavior, even if the dependence on parameter *m* was still minimal. We also investigated a new normalization factor for bEn, which ensures that bEn =1 when white Gaussian noise (WGN) is given as the input. *Conclusions*: The research improved our understanding of bubble entropy, in particular in the context of HRV analysis, and we investigated interesting details regarding the definition of the estimator.

## 1. Introduction

In a nonlinear dynamical system, the average rate of divergence of the trajectories in the state space is captured by the largest Lyapunov exponent [1]. This is also the rate at which the dynamical system *loses* information related to the initial condition or, equivalently, the rate at which information is *generated* [2]. Motivated by the objective of distinguishing chaotic systems from periodic and stochastic systems, early works of Grassberger and Procaccia [3], Takens [4], and Eckmann and Ruelle [5] proposed practical means of estimating the Kolmogorov–Sinai entropy, using a time series.

Inspired by many inconclusive results arising from practical applications of the Kolmogorov–Sinai entropy [6,7], Pincus [8] recognized that, even when only a limited amount of data is available and the system lacks stationary behavior, entropy can still be effectively employed to measure the *complexity* or the degree of *repeatability* of a time series and, indirectly, of the system that generated this series. Since then, the use of statistics quantifying the entropy rate of a time series has flourished, especially for biological series. However, real signals are inherently contaminated by noise. To deal with an arbitrary series of observations, Bandt and Pompe [9] suggested avoiding the problem altogether by measuring the entropy of the probability distribution of ordinal patterns, which, in the limit, provides an upper bound for the Kolmogorov–Sinai entropy [10].

Along this line, bubble entropy [11] was introduced to quantify the complexity of a time series by measuring the entropy of the series of swaps necessary to (bubble) sort its portions. Thus, *complexity* is intended not as a lack of similar patterns but as added diversity in the ordering of the samples across scales. As such, bubble entropy (bEn) does not directly quantify the entropy rate of the series, as Approximate Entropy (apEn) [8] and Sample Entropy (sampEn) [12] do, nor the entropy of the distribution of the possible permutations, as Permutation Entropy (pe) does. bEn measures the increase in the entropy of the series of sorting steps (swaps), necessary to order its portions of length *m*, when adding an extra element.

On the bright side, bEn is an entropy metric, with a limited dependence on parameters. This is a clear advantage over the other estimators, for which the selection of the values of the parameters is critical. The computed values, as well as the discriminating power of the estimators, depend on the parameters, and  careful estimation is essential. Taking into account that this estimation can be proven to be application- or data-dependent, the minimal dependence on parameters becomes an even more important property.

Both apEn and sampEn need to estimate two parameters: the threshold distance *r* and the embedding dimension *m*. In practice, we have accepted that the best we can do currently is to omit this step and recruit the typical values: m=1 or 2, when the length of the series permits it, and r=0.2. In a more advantageous position, bEn, similarly to pe, requires the estimation of only one parameter: the embedding dimension *m*. Not only are the degrees of freedom reduced to 1 but the remaining parameter is also an integer number, whilst the eliminated one is real.

The embedding dimension *m* ranges from 1 to a small integer value, allowing a systematic estimation of all reasonable values. On the other hand, the domain of *r* is an infinite set, making the consideration of all possible, or even reasonable, values impossible. For a detailed comparison of bubble entropy with other popular entropy metrics, please refer to [11]. In general, when tested on real data (e.g., the same datasets that are going to be considered in this paper), bEn displayed higher discriminating power over apEn, sampEn, and pe, for most values of *m*.

An analytical formulation of bEn for the autoregressive (AR) processes was recently made available [13]. This showed that, at least for this class of processes, the relation between the first autocorrelation coefficient and bEn changes for odd and even values of *m*. The authors also pointed out that the largest value of bEn did not arise for white noise but when correlations were large. While these are not issues per se, they triggered the idea that further refinements and understanding of the definition might be possible.

In this paper, we improve the comprehension of the metric, using theoretical considerations on the expected values for AR processes. We investigate a two-steps-ahead estimator of bEn, which considers the cost of ordering two additional samples. We also consider a new normalization factor that gives entropy values bEn=1 for white Gaussian noise (WGN). The rest of the paper is structured as follows. Section 3 investigates the examined normalization factor. Section 4 presents theoretical issues and simulations on the examined two-steps-ahead estimator, and Section 5 uses real HRV signals to evaluate the estimator in a real world problem. There is some discussion in Section 6, whilst the last section concludes this work.

## 2. Bubble Entropy as a Measure of Complexity

bEn embeds a given time series $x=x_{1},x_{2},\ldots ,x_{N}$ of size *N* into an *m* dimensional space, producing a series of vectors of size N−m+1:

(1) $$X_{1},X_{2},\ldots ,X_{N},\;\;\mathrm{where}\;\;X_{j}=\left(x_{j},x_{j+1},\ldots ,x_{j+m-1}\right).$$

Each vector $X_{j}$ is sorted using the *bubble sort* algorithm, and the number of swaps (inversions) required for sorting is counted.

The probability mass function (*pmf*) $p_{i}$ of having *i* swaps is used to evaluate the second-order Rényi Entropy:

(2) Hswapsm=−log∑i=0m2pi2,

while the bEn is estimated as the normalized difference of the entropy of the swaps required for sorting vectors of length m+1 and *m*:

(3) $$bEn=\left(H_{\mathrm{swaps}}^{m+1}-H_{\mathrm{swaps}}^{m}\right)/\log\left(\frac{m+1}{m-1}\right).$$

Vectors $X_{i}$ are sorted in ascending order; this is only a convention, which does not affect the time series categorization, nor the value of bEn. Indeed, if we refer to $s_{j}^{m}$ as to the number of swaps required to sort the vector $X_{j}$ in ascending order, then to sort it in descending order, $m\left(m-1\right)/2-s_{j}^{m}$ sorting steps are necessary. As such, if $p_{i}$ is the *pmf* of the swaps required for sorting all the vectors in ascending order, then sorting them in descending order will produce the *pmf* $q_{j}=p_{m(m-1)/2-j}$, which leads to an identical value of $H_{\mathrm{swaps}}^{m}$ in Equation (2).

In order to make the definition even more comprehensive, we give below an algorithmic description of the computation of bEn:

        step 1: Compute entropy in *m* dimensional space:
            step 1.1: embed the signal into *m* dimensional space
            step 1.2: for each vector, compute the number of swaps required by the bubble sort to sort it
            step 1.3: construct a series with the computed number of swaps
            step 1.4: use Rényi entropy (order 2) to compute entropy on this series
        step 2: Compute entropy in *m*+1 dimensional space
        step 3: Report the difference of entropy computed in steps 1 and 2

From the standard results in information theory [14], it is possible to cast further light on bEn. The entropy of the sum of two variables H(X+Y) is always smaller or equal to their joint entropy H(X,Y). Hence: H(X+Y)−H(X)≤H(X,Y)−H(X)=H(Y|X). The number of swaps $S^{m+1}$ required to sort a vector of length m+1 is a random variable, obtained by adding a random number of steps $S^{m}$, needed to sort a vector of length *m*, plus the extra swaps $s^{m+1}$ to take the new sample in its ordered position:

(4) $$\begin{array}{cc} S^{m} & =\sum_{k=1}^{m}s^{k}, \end{array}$$

(5) $$\begin{array}{cc} S^{m+1} & =S^{m}+s^{m+1}. \end{array}$$

Setting $X=S^{m}$ and $Y=s^{m+1}$ in the relation just derived, and remembering that the mutual information: I(X;Y)=H(Y)−H(Y|X), then:

(6) $$H\left(S^{m+1}\right)-H\left(S^{m}\right)\leq H\left(s^{m+1}|S^{m}\right)=H\left(s_{m+1}\right)-I\left(S^{m};s_{m+1}\right)$$

or:

(7) $$bEn\;\log\left(\frac{m+1}{m-1}\right)=H_{\mathrm{swaps}}^{m+1}-H_{\mathrm{swaps}}^{m}\leq H\left(s_{m+1}\right)-I\left(S^{m};s_{m+1}\right).$$

bEn is, therefore, limited from above by the entropy of the number of swaps required to add the extra element in the vector, reduced by the information already carried by the number of swaps performed before.

In the following, we will use the term $bEn_{+\;1}$, instead of simply bEn, which has been used until now. We want to make a clear distinction between this definition and an alternative one, which will be considered later in this paper.

## 3. On the Investigation of the Normalization Factor

The normalization factor $\log\left(\frac{m+1}{m-1}\right)$ in Equation (3) is given by the difference in the maximum swaps quadratic entropy, which is $U_{\mathrm{swaps}}^{m}=\log\left[\frac{(m-1)m}{2}+1\right]$ for the embedding dimension *m* and $U_{\mathrm{swaps}}^{m+1}=\log\left[\frac{m(m+1)}{2}+1\right]$ for embedding dimension m+1, when neglecting the term +1 in the logarithms. This term corresponds to the *no swaps performed* state and the simplification contributes toward a more elegant definition, without significant influence on the numerical value, especially for larger values of *m*.

Common definitions of entropy present maximum entropy values when WGN is given as the input. However, in our previous work [13], we showed that $bEn_{+\;1}$ is not maximal for WGN. Signals produced by the AR model with large and positive one-step autocorrelation tended to require a broader range of swaps than WGN, and the uniform distribution had the largest entropy among all discrete probability mass functions. We will come back to this observation, after we introduce one more definition: $bEn_{+\;1}^{*}$.

The analytical value of $bEn_{+\;1}$ for WGN can be obtained in a simpler fashion than that described in [13]. The probability generating function of the number of inversions required to sort a random permutation of *m* numbers [15] is given by:

(8) $$h_{m}\left(z\right)=\frac{1}{m!}\prod_{k=0}^{m-1}\sum_{j=0}^{k}z^{j}=\frac{1\left(1+z\right)\cdots \left(1+z+\cdots +z^{m-1}\right)}{m!}.$$

Indeed, according to Equation (5), the total number of swaps required for a WGN is a random variable obtained as the sum of *m* independent discrete uniform random variables with support [0,k] and k=0…m−1. Thus, given *k* samples, which are already sorted, a new random value in position k+1 requires any from 0 to *k* inversions, each with the probability 1/(k+1), to be swapped into the correct position. The probability generating function for the number of additional inversions is: $s_{k+1}\left(z\right)=\left(1+z+z^{2}+\cdots +z^{k}\right)/\left(k+1\right)$. The probability generating function of the total number of inversions $h_{k+1}\left(z\right)$ is given as the product of the additional permutations and $h_{k}\left(z\right)$, where $h_{1}\left(z\right)=1$:

(9) $$h_{k+1}\left(z\right)=s_{k+1}\left(z\right)h_{k}\left(z\right)=\frac{1+z+z^{2}+\cdots +z^{k}}{k+1}h_{k}\left(z\right).$$

As the number of permutations with no inversions is 1, we can obtain Equation (8).

Then, from the definition of the probability generating function, the *pmf* $p_{i}$, having *i* swaps, is the coefficient of the *i*-order term in the polynomial $h_{m}\left(z\right)$, or:

(10) $$p_{i}=\frac{1}{i!}{\left(\frac{d^{i}\;h_{m}\left(z\right)}{d\;z^{i}}\right)}_{z=0}=\frac{h_{m}^{\left(i\right)}\left(0\right)}{i!}.$$

The entropy of the series of swaps, for WGN, is a growing function with *m*:

(11) Wswapsm=−log∑i=0m2hm(i)(0)i!2,

and the bEn of a WGN:

(12) $$bEn_{\;+1}^{\mathrm{WGN}}=\;\left(W_{\mathrm{swaps}}^{m+1}-W_{\mathrm{swaps}}^{m}\right)/\log\left(\frac{m+1}{m-1}\right).$$

Having now the values of entropy of the series of swaps for a WGN, for any value of *m*, we define, as $bEn_{+\;1}^{*}$, the ratio:

(13) $$bEn_{\;+1}^{*}=\frac{bEn_{\;+1}}{bEn_{\;+1}^{\mathrm{WGN}}}=\frac{H_{\mathrm{swaps}}^{m+1}-H_{\mathrm{swaps}}^{m}}{W_{\mathrm{swaps}}^{m+1}-W_{\mathrm{swaps}}^{m}}.$$

In other words, $bEn_{+\;1}^{*}$ is the difference of the entropy in the dimensional spaces m+1 and *m*, normalized with the difference of the entropy of WGN in these spaces.

**Figure 1:**
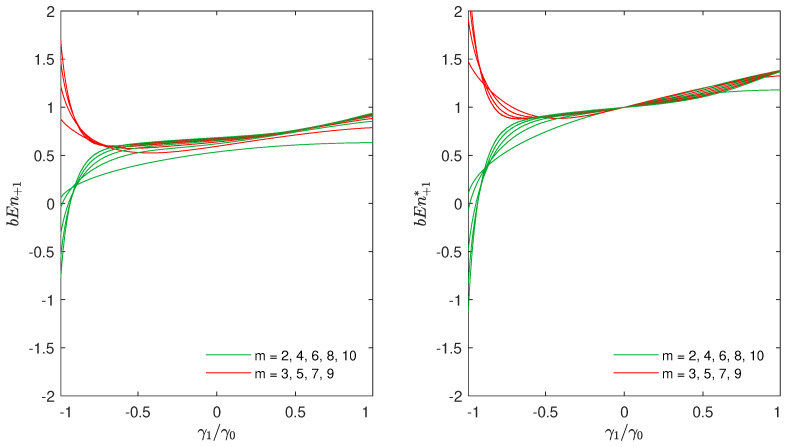
Numerical estimates of $bEn_{+\;1}$ and $bEn_{+\;1}^{*}$ for sequences generated by the AR process of order 1, $x\left[n\right]=-a_{1}x\left[n-1\right]+w\left[n\right]$, where $a_{1}\in \left[-1,1\right]$, and w[n] is a WGN with zero mean and variance $\sigma_{w}^{2}=1$. $\gamma_{k}$ is the correlation at lag *k*, and $\gamma_{1}/\gamma_{0}=-a_{1}$. In the left subfigure, the definition of $bEn_{+\;1}$ was used in the simulations, whilst, in the right subfigure is the definition of $bEn_{+\;1}^{*}$. In the right panel, bubble entropy is always one for WGN.

Let us cover the benefits of using this definition. In Figure 1, we generate a series from the AR process of order 1:

(14) $$x\left[n\right]=-a_{1}x\left[n-1\right]+w\left[n\right],$$

where $a_{1}\in \left[-1,1\right]$, and w[n] is WGN with the mean μ=0 and variance $\sigma_{w}^{2}=1$. The correlation at lag *k* is $\gamma_{k}=E\left[x[n]x[n-k]\right]$ and $\gamma_{1}/\gamma_{0}=-a_{1}$. Numerical estimates of $bEn_{+\;1}^{*}$ were computed over 1000 Monte Carlo simulations with a series of $N=10^{5}$ samples.

In the subfigure on the left hand side of Figure 1, we used the definition of $bEn_{+\;1}$, as we did in [13]. In the subfigure on the right hand side of the figure, we can see the difference, in the same experiment, when $bEn_{+\;1}^{*}$ was used. Please note that, with the definition of $bEn_{+\;1}^{*}$, the entropy for WGN ($\gamma_{1}/\gamma_{0}=-a_{1}=0$) is equal to 1 for all values of *m*.

Since, this is an important property, we found the normalization proposed in Equation (13) to be more appropriate than the one in Equation (3), which was used until now. In fact, values of bubble entropy computed with this normalization can be used to compare different processes and/or to put them in relation with WGN (a bEn larger than one means that the *pmf* of the number of swaps becomes more “spread out” than for a WGN, when *m* increases). This normalization further reduces the dependence on the residual parameter *m*, as nearly identical values of bEn for $\gamma_{1}/\gamma_{0}>0$ and m>2 in the right panel of Figure 1 show.

The value of $W_{\mathrm{swaps}}^{m}$ in Equation (11) is exact; however, its computation is challenging for large values of *m*, due to the growth of the factorial term. However, in this situation, when *m* is large, for the central limit theorem, the discrete *pmf*$p_{i}$ converges in distribution to a normal probability density function (*pdf*), with the same mean and variance. Due to the symmetry of the *pmf*, the mean of $p_{i}$ is $\mu_{\mathrm{swaps}}=m\left(m-1\right)/4$, which can also be obtained as $\mu_{\mathrm{swaps}}={\left[dh_{m}\left(z\right)/dz\right]}_{\left(z=1\right)}$. The variance can be obtained from the probability generating function:

(15) $$\sigma_{\mathrm{swaps}}^{2}={\left\{\frac{d^{2}\;h_{m}\left(z\right)}{d\;z^{2}}+\frac{d\;h_{m}\left(z\right)}{d\;z}-{\left[\frac{d\;h_{m}\left(z\right)}{d\;z}\right]}^{2}\right\}}_{\left(z=1\right)}=\frac{m(m-1)(2m+5)}{72}.$$

Alternatively, $\sigma_{\mathrm{swaps}}^{2}$ can be computed by observing that the variance of the discrete uniform distribution has the probability generating function $s_{k}\left(z\right)=\left(1+z+z^{2}+\cdots +z^{k-1}\right)/k$ is $(k^{2}-1)/12$. The total number of inversions is the sum of *m* independent random variables and, as a consequence, $\sigma^{2}$ is the sum of the single variances:

(16) $$\sigma_{\mathrm{swaps}}^{2}=\sum_{k=0}^{m-1}\frac{k^{2}-1}{12}=\frac{m(m-1)(2m+5)}{72}.$$

The Rényi entropy of order 2 (or quadratic entropy) of a normal *pdf* is $\frac{1}{2}\log\left(4\pi \sigma^{2}\right)$, where $\sigma^{2}$ is its variance. When *m* is large, we can then approximate the entropy of the series of swaps for a WGN with:

(17) $$W_{\mathrm{swaps}}^{m}\approx \frac{1}{2}\log\left[\pi \frac{m(m-1)(2m+5)}{18}\right].$$

In our numerical tests, the approximation holds well for values of m≥30, where the error is already smaller than 1${}^{o}\;/{\;}_{\mathrm{oo}}$. For smaller values of *m*, Equation (11) should be employed instead.

## 4. On the Investigation of the Two-Step-Ahead Estimator of Bubble Entropy

Let us stay for a while at Figure 1. In both subfigures of Figure 1, for anti-persistent noise, i.e., when $\gamma_{1}/\gamma_{0}$ approaches −1, the values of entropy become negative for even values of *m*, whilst they are largely positive for odd values of *m*. This is another issue that gives us motivation for further investigation.

**Figure 2:**
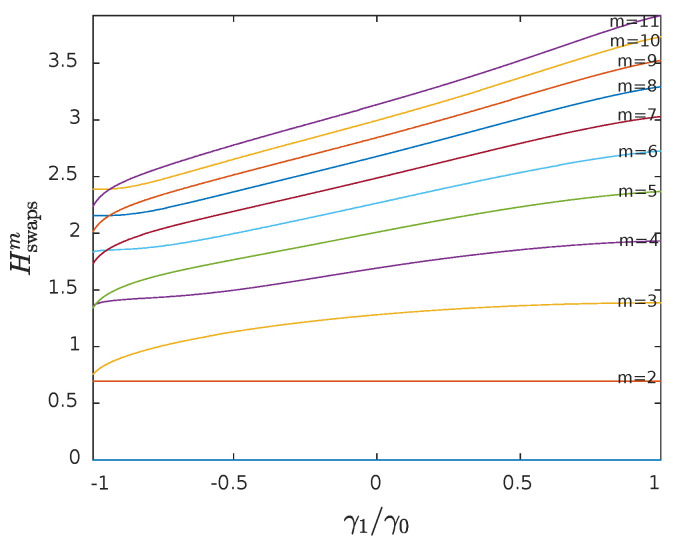
The average numerical values of $H_{\mathrm{swaps}}^{m}$ for m=2,…,11 (bottom to top), using the same simulations described in Figure 1.

In Figure 2, the average numerical values of $H_{\mathrm{swaps}}^{m}$ for m=2,…,11 are presented, using the same simulations described in Figure 1. The lines at the lower part of the figure correspond to lower values of *m*. When *m* is even and $\gamma_{1}/\gamma_{0}$ is approaching to −1, we can observe that $H_{\mathrm{swaps}}^{m+1}<H_{\mathrm{swaps}}^{m}$ is a possible condition, something that results in negative values ($bEn_{+\;1}^{*}$<0).

**Figure 3:**
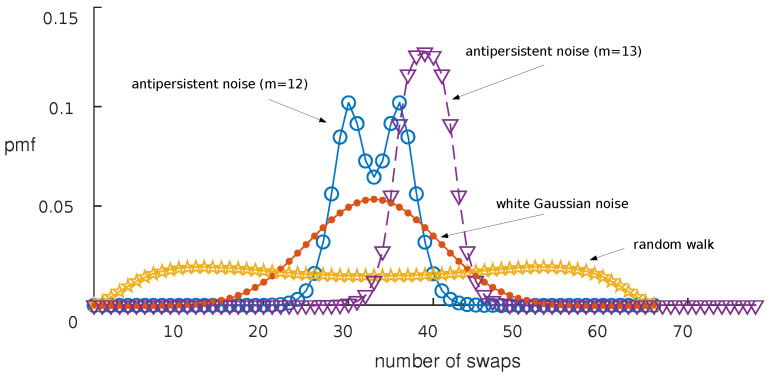
Probability mass functions of the number of swaps sorting series with $\gamma_{1}/\gamma_{0}=-1$, $\gamma_{1}/\gamma_{0}=0$, $\gamma_{1}/\gamma_{0}=1$.

Even though this is not a problem per se, to further analyze this observation, we plotted the *pmf*s in Figure 3. In this figure, four *pmf*s obtained from the 1st order AR model, averaging over 100 Monte Carlo runs, with the series length $N=10^{5}$, are displayed. The four *pmf*s express the number of swaps necessary to sort: (a) an m=12 long sequence with $\gamma_{1}/\gamma_{0}=0$, that is WGN; (b) an m=12 long sequence with $\gamma_{1}/\gamma_{0}=1$, that is a random walk; (c) an m=12 long sequence with $\gamma_{1}/\gamma_{0}=-1$, that is an anti-persistent noise; and (d) an m=13 long sequence with $\gamma_{1}/\gamma_{0}=-1$ depicting again anti-persistent noise.

It is interesting to note that, given the fact that WGN does not display the largest value of $H_{\mathrm{swaps}}^{m}$, its *pmf* is more concentrated around the average $\mu_{\mathrm{swaps}}=m\left(m-1\right)/4$ than that of the random walk. More interesting is the observation that the *pmf* of the anti-persistent noise is further concentrated around the mean. In the case in which m=12, two peaks appear at $\mu_{\mathrm{swaps}}\pm m/4$.

This is something that occurs generally for even values of *m*. In fact, sorting the degenerate limit sequence 1,−1,…,−1 always requires m/2 swaps more than sorting the series −1,1,…,1. On the contrary, for odd values of *m*, there is always only a single peak at $\mu_{\mathrm{swaps}}$, since sorting the series 1,−1,…,1 and −1,1,…,−1 has the same cost: (m−1)(m+1)/8. To illustrate this single peak, the *pmf* for $\gamma_{1}/\gamma_{0}=-1$ and m=13 is also included in the figure. The larger spread around the mean, due to the two peaks appearing for even values of *m*, explains why $H_{\mathrm{swaps}}^{m+1}<H_{\mathrm{swaps}}^{m}$ is possible.

The above conclusions led us to define a two-steps-ahead estimator for bubble entropy by setting:

(18) $$bEn_{\;+2}^{*}=\frac{H_{\mathrm{swaps}}^{m+2}-H_{\mathrm{swaps}}^{m}}{W_{\mathrm{swaps}}^{m+2}-W_{\mathrm{swaps}}^{m}}.$$

**Figure 4:**
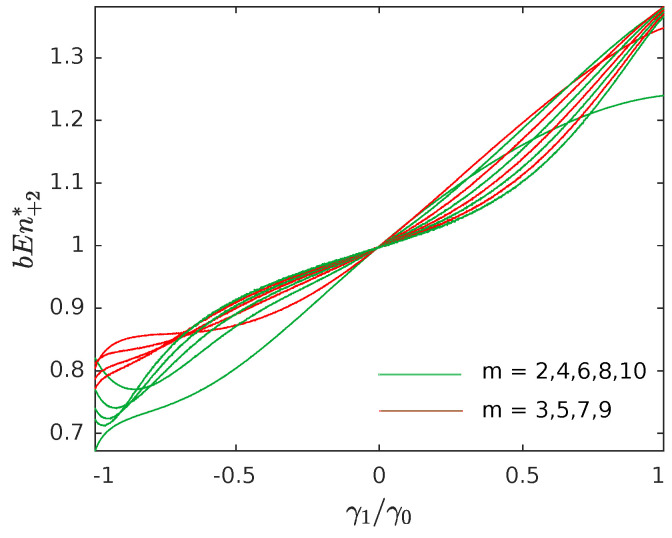
The average numerical values of $bEn_{+\;2}^{*}$, using the same simulations (and legend) in Figure 1.

In other words, instead of computing the difference in entropy between spaces with dimensions *m* and m+1, we consider the variation between the spaces *m* and m+2 (hence the pedix +2 instead of +1). This consideration allows us to compare the number of swaps required to sort the vectors belonging to odd dimensional spaces (*m* odd) with odd dimensional spaces (m+2 odd) and even dimensional spaces (*m* even) with even dimensional spaces (m+2 even), eliminating the asymmetry detected between odd and even spaces. It also leads to positive and growing entropy values, as shown in Figure 4, in contrast to the behavior observed in Figure 1.

To rationalize the relation between $bEn_{+\;1}^{*}$ and $bEn_{+\;2}^{*}$, we notice that:

(19) $$\left(H_{\mathrm{swaps}}^{m+2}-H_{\mathrm{swaps}}^{m+1}\right)\;+\;\left(H_{\mathrm{swaps}}^{m+1}-H_{\mathrm{swaps}}^{m}\right)=H_{\mathrm{swaps}}^{m+2}-H_{\mathrm{swaps}}^{m}.$$

Indeed:

(20) $$\begin{array}{cc} \min\left(H_{\mathrm{swaps}}^{m+2}-H_{\mathrm{swaps}}^{m+1},H_{\mathrm{swaps}}^{m+1}\right.\;- & \left.H_{\mathrm{swaps}}^{m}\right)\;\leq \;\frac{H_{\mathrm{swaps}}^{m+2}-H_{\mathrm{swaps}}^{m}}{2}\leq \\ \leq & \max\left(H_{\mathrm{swaps}}^{m+2}-H_{\mathrm{swaps}}^{m+1},H_{\mathrm{swaps}}^{m+1}-H_{\mathrm{swaps}}^{m}\right) \end{array}$$

and, in practical applications, where empirically $H_{\mathrm{swaps}}^{m}$ is found to decrease with *m*:

(21) $$H_{\mathrm{swaps}}^{m+2}-H_{\mathrm{swaps}}^{m+1}\leq \frac{H_{\mathrm{swaps}}^{m+2}-H_{\mathrm{swaps}}^{m}}{2}\leq H_{\mathrm{swaps}}^{m+1}-H_{\mathrm{swaps}}^{m}.$$

When *m* is large, the two bracketing values approach and:

(22) $$H_{\mathrm{swaps}}^{m+1}-H_{\mathrm{swaps}}^{m}\approx \frac{H_{\mathrm{swaps}}^{m+2}-H_{\mathrm{swaps}}^{m}}{2},$$

or, equivalently, for a WGN:

(23) $$W_{\mathrm{swaps}}^{m+1}-W_{\mathrm{swaps}}^{m}\approx \frac{W_{\mathrm{swaps}}^{m+2}-W_{\mathrm{swaps}}^{m}}{2}.$$

While Equation (23) is exact for a stationary process in the limit m→+∞, we empirically verified that it holds sufficiently well for “practical” values of *m*. For example, the difference is smaller than 5% when m>8.

Now, taking the ratio side by side of Equations (22) and (23), we derive that:

(24) $$bEn_{\;+2}^{*}\approx bEn_{\;+1}^{*}.$$

Therefore, the two estimators provide estimates that are quantitatively equivalent (e.g., both are 1 for a WGN).

## 5. Experimental Analysis

In order to support our theoretical considerations, we tested our observations on real HRV signals as well, obtained from Physionet [16]. The first data set is the *Normal Sinus Rhythm (NSR) RR Interval Database* (nsr2db). This database includes beat annotations for 54 long-term ECG recordings of subjects in normal sinus rhythm (30 men, aged 28.5 to 76, and 24 women, aged 58 to 73). The original ECG recordings were digitized at 128 samples per second, and the beat annotations were obtained by automated analysis with manual review and correction.

The second data set is the *Congestive Heart Failure (CHF) RR Interval Database* (chf2db). This database includes beat annotations for 29 long-term ECG recordings of subjects aged 34 to 79 years, with congestive heart failure (NYHA classes I, II, and III). The subjects included eight men and two women; gender was not known for the remaining 21 subjects. The original ECG recordings were digitized at 128 samples per second, and the beat annotations were also obtained by automated analysis with manual review and correction.

The HRV series were obtained from the beat annotation files. Only normal-to-normal beat intervals were considered. To reduce the impact of artifacts on the estimates of the metrics, we removed all NN intervals, which differed more than 30% from the previous NN interval.

**Figure 5:**
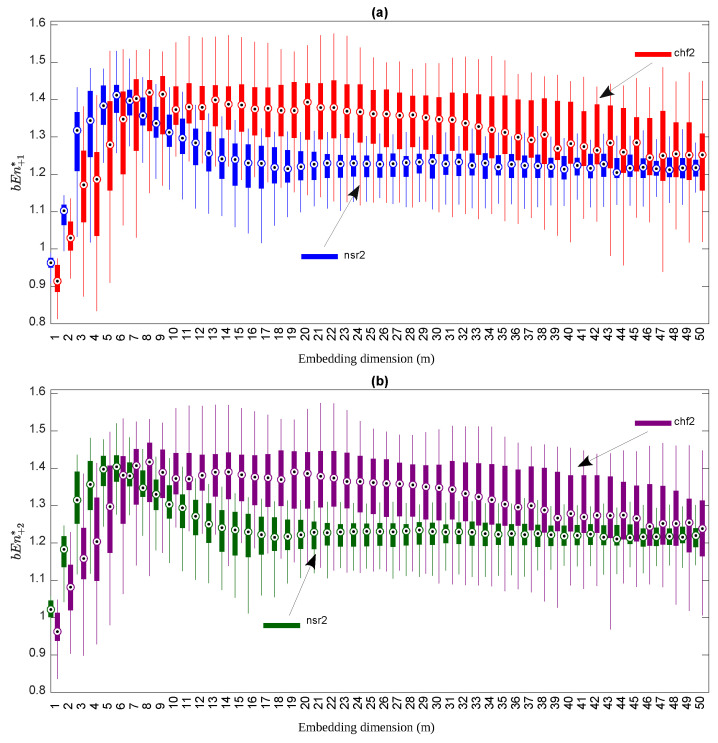
Box-plots for the values of $bEn_{\;+1}^{*}$ (**a**) and $bEn_{\;+2}^{*}$ (**b**) for the controls (nsr2) and congestive heart failure patients (chf2), while *m* ranges in m=1,…,50.

Our target was to compare the discriminating power of the two definitions of bubble entropy, $bEn_{+\;1}^{*}$ and $bEn_{+\;2}^{*}$, to separate the two groups of subjects. We used *p*-values (the Mann–Whitney U test) as a criterion. We computed the bubble entropy for $bEn_{+\;1}^{*}$ and $bEn_{+\;2}^{*}$ for values of *m* ranging from m=1 to m=50. In Figure 5, we present the box plots for both $bEn_{+\;1}^{*}$ (the top subfigure of Figure 5) and $bEn_{+\;2}^{*}$ (the bottom subfigure or Figure 5).

**Figure 6:**
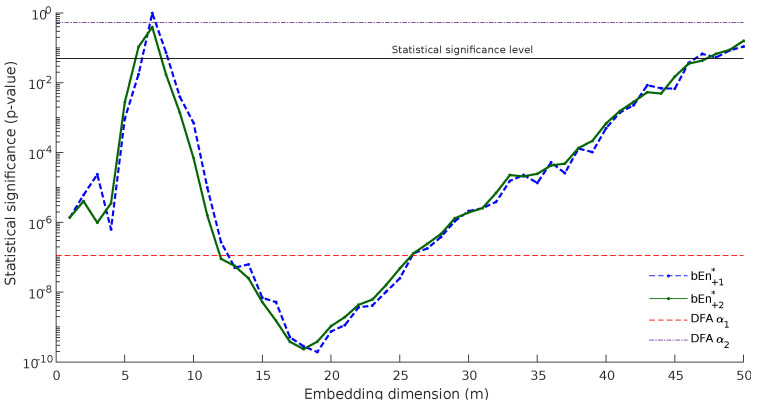
Comparison using the discrimination between the control and congestive heart failure patients (nsr2db and chf2db databases). Blue line depict *p*-values computed for the $bEn_{+\;1}^{*}$ estimator and green lines depict $bEn_{+\;2}^{*}$.

For the simulations and as expected from the theoretical considerations, the values of $bEn_{+\;1}^{*}$ and $bEn_{+\;2}^{*}$ were similar. Normal subjects displayed a bEn that was larger than CHF patients for small values of *m* (≤5), while it became smaller when *m* is large. The results of the statistical test are depicted in Figure 6. The blue dashed line represents *p*-values computed with $bEn_{+\;1}^{*}$, whilst the green line shows the corresponding *p*-values for $bEn_{+\;2}^{*}$. Please note that this is a log plot. There are two main conclusions from this graph:

- The *p*-values computed by $bEn_{+\;2}^{*}$ were smaller than those computed with $bEn_{+\;1}^{*}$, especially in the region of 10<m<20, where the method appeared to succeed with better classification, which improved as *m* increased.
- $bEn_{+\;2}^{*}$ presented a smoother behavior, especially in the same area: 10<m<20. This is in accordance with the our main hypothesis that the method behaves in a different way for odd and even values of *m*.

In order to have a better sense of the discriminating power of the method, we marked on Figure 6 the statistical significance level (blue solid line at 0.05) and the *p*-values computed by Detrended Fluctuation Analysis (DFA) [17], index DFA$\alpha_{1}$, and DFA$\alpha_{2}$. In the context of HRV series obtained from 24-h Holter recordings, the slope DFA$\alpha_{1}$ is typically found to be in the range $0.9<\alpha_{1}<1.2$ for normal subjects, DFA$\alpha_{1}>1.33$ for CHF patients and DFA$\alpha_{1}>1.5$ for subjects who survived a myocardial infarction [18]. Bubble entropy always presents significantly better categorization than DFA$\alpha_{2}$ and better categorization than DFA$\alpha_{1}$ for 12<m<25.

**Figure 7:**
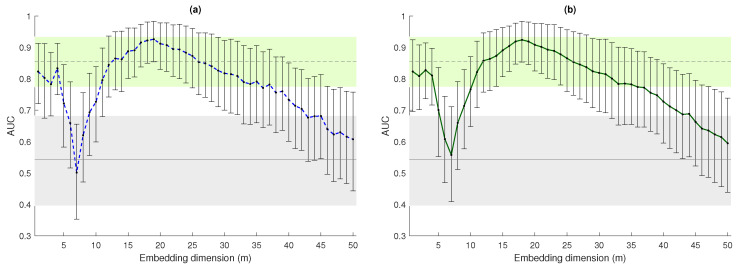
Area-Under-the-ROC-Curve (AUC) values when using $bEn_{+\;1}^{*}$ (blue sketched line, **a**) and $bEn_{+\;2}^{*}$ (green solid line, **b**) to distinguish between subjects in the nsr2db and chf2db databases. The 95% confidence intervals, also reported, were obtained with 1000 bootstrap resamplings. The gray sketched and solid lines are the AUC values for DFA$\alpha_{1}$ and DFA$\alpha_{2}$, respectively, as well as their 95% confidence intervals (shaded areas).

For completeness, in Figure 7, we present the Area-Under-the-ROC-Curve (AUC) computed when using either $bEn_{+\;1}^{*}$ and $bEn_{+\;2}^{*}$ to discrimnate the two populations. We also included the 95% confidence intervals, computed using bootstrap (1000 iterations), to compensate for the low dimension of the two populations. Coherent conclusions can be drawn from these plots. The results in the figure confirm that bEn discriminated the HRV series of normal subjects and CHF patients when *m* was small (m≤4) with an AUC comparable to DFA$\alpha_{1}$. We speculate that the characteristics of the signal they are picking up might be the same.

Then, bEn shows a very large AUC also for about 15≤m≤25. While DFA$\alpha_{2}$ detects the long-term memory characteristics of the series, it does not distinguish between the two populations, so the features detected by bEn might be different. Of note, the definition of bEn permits the exploration of ranges of *m*, which are not typically accessible with other entropy measures, such as ApEn or SampEn (*m* was rarely larger then 3, due to convergence issues) or Permutation Entropy (typical values of *m* are smaller than 10 as the factorial terms increase very quickly).

## 6. Discussion

Even though this paper examines alternatives in the definition of bubble entropy, we took the opportunity to summarize in this last section some conclusions on the comparison of bubble entropy with other popular definitions, especially in the *m*-dimensional space.

Definitions in the *m*-dimensional space extract more sensitive information compared with the more classic definitions in 1-dimensional space. They not only exploited the values of the samples but also their order. If, for example, we shuffle the samples of a time series, the values of both Shannon and Rényi entropy will be unaffected. Due to their increased sensitivity, definitions in *m*-dimensional space became quite popular and are valuable tools in entropy analysis.

Approximate and sample entropy are almost always used in research work involving entropy analysis, especially in biological signals. We chose to compare bubble entropy with those widely accepted estimators. We already discussed, in the introductory section, the main advantages of bubble entropy analysis against approximate and sample entropy, emphasizing the minimal dependence on parameters. We also discussed the discriminating power, not only for approximate and sample entropy but also for permutation entropy, an entropy metric that inspired bubble entropy.

As stated above, it was not the objective of this paper to make a comparison between bubble entropy and other entropy metrics. For this, we refer the interested reader to our previous work [11]. However, to add perspective, we included into Figure 6 and Figure 7 the values of DFA $\alpha_{1}$ and $\alpha_{2}$. Detrended fluctuation analysis does not quantify entropy-related metrics, but it is a “fractal” method that proved effective in distinguishing healthy subjects from CHF patients [18]. The values of DFA$\alpha_{2}$ are also related to the Hurst exponent for long-term memory processes, as well as bubble entropy in [19].

## 7. Conclusions

The contributions of this paper are two-fold. First, we introduced an alternative normalization factor for bubble entropy, based on the theoretical value of bubble entropy, when WGN is given as input. With the new normalization factor, the entropy of WGN is always equal to 1, for every value of *m*. While theoretically interesting, this consideration does not affect the discriminating power of the method, since this is only a common scalar value.

The second contribution of this paper is the investigation of a two-steps-ahead estimator for bubble entropy. Since we showed that (rarely) is there (when the series are strongly anti-correlated) an asymmetry in the cost of bubble sorting between odd and even dimensional spaces, we computed the bubble entropy to compare the entropy between embedding spaces with odd dimensions or between embedding spaces with even dimensions. The advantage of this approach was illustrated with experiments with both simulated and real HRV signals.

The simulated WGN signals showed that, for anti-persistent noise, where the asymmetry between spaces with odd and even dimensions was maximally expressed, bubble entropy presented similar values for all values of *m*, in contrast to the initial approach, where the entropy values for anti-persistent noise were significantly different between successive values of *m*. Theoretically, this consideration improves the discriminating power of the method, even though conditions similar to strongly anti-persistent noise are not often found in HRV signals.

For completeness, we performed experiments with real HRV signals, which were publicly available and are widely used. The experiments showed that both examined definitions showed comparable discriminating power between the NSR and CHF signals. However, for values of 10<m<20, the two-steps-ahead estimator presented slightly higher statistical significance and more regular behavior, with a smoother difference between successive values of *m*.

The research increased our understanding of bubble entropy, in particular in the context of HRV analysis. The two-steps-ahead estimator, while a minor refinement, should not be ignored in future research directions.

## Data Availability

Not applicable.

## Abbreviations

The following abbreviations are used in this manuscript:

AR	AutoRegressive
HRV	Heart Rate Variability
WGN	White Gaussian Noise
NSR	Normal Sinus Rhythm
CHF	Congestive Heart Failure
ECG	Electrocardiogram
